# Immune responses following intrapleural administration of oncolytic SEPREHVIR in patients with malignant pleural mesothelioma

**DOI:** 10.1186/2051-1426-3-S2-P335

**Published:** 2015-11-04

**Authors:** Kirsty Learmonth, Lynne Braidwood, Penella Woll, Joe Conner

**Affiliations:** 1 Virttu Biologics, Glasgow, UK; 2 University of Sheffield/Sheffield Teaching Hospitals, Sheffield, UK

## Background

Malignant pleural mesothelioma (MPM) remains a major challenge, with limited therapeutic options. Multifocal intrapleural disease can cause disabling symptoms of pain and breathlessness, in the absence of distant metastases, so an intrapleural treatment approach is attractive. SEPREHVIR (HSV1716) is a mutant oncolytic herpes simplex virus type 1 deleted in the gene encoding the neurovirulence factor ICP34.5. Mutants lacking ICP34.5 are selectively replication competent in cancer cells and induce anti-tumour immune responses. Clinical studies with SEPREHVIR have been completed in adult glioma, melanoma, H&NSCC, and studies are ongoing in both pediatric CNS and non-CNS solid tumors and MPM. In total, 92 patients have received SEPREHVIR via intratumoral, loco-regional, and intravenous administration and it is well-tolerated with no shedding in patients. SEPREHVIR selectivity for replication only in tumour cells and intimations of efficacy and immuno-stimulatory potential have been demonstrated.

## Methods

A Phase I/IIa trial to determine the safety and potential for efficacy of SEPREHVIR given intrapleurally to patients with MPM is currently ongoing. Patients receive 1x10^7^iu SEPREHVIR through their pleural catheter on one, two or four occasions a week apart, in three separate patient cohorts. To date 9 patients have been treated, 3 in each cohort and SEPREHVIR has been well-tolerated with few adverse events in any patients. Pleural fluid and plasma samples have been collected pre- and post treatment and analysed to assess patient responses to SEPREHVIR administration. Pleural fluids have been analysed by ELISA for cytokines/chemokines and were rich in IL-6, IL-8, MIG and VEGF.

## Results

Robust Th1 responses with increased IFN-α, IP-10, MIG and TNFα were observed in most patients after SEPREHVIR administration with IL-2, IL-10 and IL-12 responses in patients receiving 4 doses. IL-1α, IL-4, IL-21 and IFN-α were not detected pre-treatment and, along with IL8, showed minimal responses to SEPREHVIR administration.

Analysis of plasma samples indicated strong anti-HSV IgG responses post SEPREHVIR administration, particularly after 2 and 4 doses and the presence of anti-HSV IgG in pleural fluids. Despite this, HSV DNA was detected in the pleural fluids of most patients and in some persisted for at least two weeks post-administration. Interestingly, in most patients, there was a novel anti-tumour IgG response as detected by immunoblotting against extracts from MPM cell lines.

## Conclusions

Thus, our trial demonstrates that oncolytic SEPREHVIR has immunotherapeutic potential capable of inducing novel anti-tumour immune responses in patients and further studies are ongoing. This also confirms pre-clinical studies that clearly demonstrated Seprehvir's immunotherapeutic mode of action.

